# Does Religion Influence the Motivations of Future Healthcare Professionals to Volunteer During the COVID-19 Pandemic in Poland? An Exploratory Study

**DOI:** 10.1007/s10943-021-01231-8

**Published:** 2021-04-02

**Authors:** Jan Domaradzki, Dariusz Walkowiak

**Affiliations:** 1 Department of Social Sciences and Humanities, Poznan University of Medical Sciences, Rokietnicka 7, St, 60-806 Poznań, Poland; 2 Department of Organization and Management in Health Care, Poznan University of Medical Sciences, Poznań, Poland

**Keywords:** COVID-19 pandemic, Religion, Volunteering, Future healthcare professionals

## Abstract

This study was designed to determine the relation between religion and the motivations of future healthcare professionals to volunteer during the COVID-19 outbreak in Poland**.** Data were collected from 417 healthcare students via an online questionnaire. The results indicated that although students’ religiosity was not a significant predictor of volunteering during the pandemic, it played a key role in determining their motivations to join the fight against COVID-19. There was a significant positive relationship between students’ religiosity and their eagerness to commit for the sake of the community rather than for personal or egoistic motives.

## Introduction

Ever since sociology has been established as a scientific discipline, religion has been studied as a basic social institution, a central phenomenon of social life and an influential core for many cultures. Religiosity can also be an essential factor shaping physicians’ attitude towards patients (Pawlikowski et al., 2012; Wenger & Carmel, 2004). Unsurprisingly, religion has been defined as an important predictor of volunteering, and it is now well documented that both religious beliefs and practices influence volunteers’ motivations. Moreover, while research shows that the importance of helping others is more common among religious people, it has also been found that members of religious congregations volunteer more frequently and dedicate more hours to voluntary service (Abuiyada, 2018; Ariza-Montes et al., 2018; DeAngelis et al., 2016; Einolf, 2011; Fényes, 2015; Hill & Dulk, 2013; Hustinx et al. 2015; Merino, 2013; Monsma, 2007; Paxton et al. 2014; Wilson & Janoski, 1995; Yeung, 2018). For example, Johnston (2013) reported that religion is strongly associated with volunteering for both religious and non-religious institutional forms of volunteering and that it increases the likelihood of volunteering in adult life. Also, Niebuur et al. (2018) and Herzog et al. (2020) reported that religious beliefs, affiliation, comfort in religion and religious salience are all associated with volunteering rates. Recent research conducted among Australian university students and the general population found that religious beliefs increased personal volunteering, while religious affiliation and service attendance increased pro-social motivations for volunteering (Petrovic et al., 2020). Similarly, religious practices had a positive effect on volunteering in Eastern Europe (Voicu & Voicu, 2009).

However, although many scholars have highlighted the importance of volunteering to building and maintaining a civil society, civic participation in Poland, due to historical and political reasons, is much lower than in Western countries, and volunteering, especially among Polish students, is a relatively new activity. Consequently, estimates show that only 6% of Poles engaged in volunteering in 2010 and only 11% in 2011 (Centrum Badania Opinii Społecznej, 2011a, b). At about the same time, however, it was also reported that Polish volunteers were characterised by their religiosity and attachment to the institutional Church and that volunteers’ engagement rests mainly on religious and moral motivations (Szluz, 2012; Szymańska-Palaczyk, 2016; Główny Urząd Statystyczny, 2017). Moreover, according to the Centre for Public Opinion Research, while the number of volunteers who practice their religion regularly increased significantly after 2010, those who practice religion less frequently, or are religiously indifferent, decreased (Centrum Badania Opinii Społecznej, 2012).

Contemporaneously, because secularisation resulted in the drastic decline of religious practices in Europe (Davies, 2006), it has also changed the conditions of and motivations for volunteering (Jaronowska, 2017; Olczyk, 2015; Petrovic et al., 2020; Voicu & Voicu, 2009). Indeed, research shows that in many countries, including Poland, modernisation, globalisation, Europeanisation and economic development have weakened the positive relationship between religion and volunteering. Moreover, as people lose their religious identity, they perceive voluntary service in a more secular fashion, i.e. as a form of social activism, and not as a religious or moral duty in the form of an apostolate (Voicu & Voicu, 2009; Handy et al., 2010; Grönlund, 2012; Fényes & Pusztai, 2012; Centrum Badania Opinii Społecznej, 2012; Sawicki, 2014; Fényes, 2015; Lubrańska & Zawira, 2017). For example, while earlier studies have reported that 60.7% of the adult volunteers in Poland were motivated by moral, religious or political beliefs (Szluz, 2012), a recent study conducted among Polish students found that only 4% were driven strictly by religious motivations, with most more concerned about having a personal feeling of satisfaction from helping others (81%), wanting to gain professional experience (67%), help others (46%) or to meet new people (39%) (Jaronowska, 2017).

This is important, especially during the current health crisis caused by the COVID-19 outbreak, because volunteering constitutes the chief example of civic consciousness and responsibility (Chawłowska et al., 2021; Drexler et al., 2020; Gallagher & Schleyer, 2020; Gouda et al., 2020; Miller et al., 2020; Soled et al., 2020; Thomson & Lovegrove, 2020). This is especially so in countries like Poland, which struggle with the problem of a decreasing number of health professionals (Eurostat, 2020a), or in countries that have experienced care staff shortages during the COVID-19 pandemic (Rasmussen et al., 2020; Stokes, 2020). Thus, ever since the COVID-19 struck and an epidemic was announced by the Polish government, medical universities have started encouraging future healthcare professionals to volunteer and support the fight against the pandemic. Consequently, even though all universities in the country were closed, lectures were moved online and all medical students were pulled out of the hospital’s, thousands of students of medicine, nursing, pharmacy, medical analytics, medical rescue and physiotherapy volunteered and started helping in local hospitals, emergency units, hospital pharmacies, sanitary-epidemiological stations, the university’s diagnostic laboratory and local call centres.

Thus, this paper aims to describe the impact of religiosity on the motivations to volunteer among future healthcare professionals during the COVID-19 outbreak in Poland.

## Methods

This study was conducted between 5 May and 30 June 2020 among students enrolled in Poznan University of Medical Sciences (PUMS), Poland using an online questionnaire posted on an online platform. The questionnaire consisted of three sections, the first asked questions regarding students’ demographic characteristics, the second section gathered information on students’ reactions to the pandemic and anxieties related to voluntary service, and the last section included questions regarding students’ motivations to volunteer. In this part, respondents were asked to rank on a scale of 1–5 the reasons they volunteered, and the mean values were computed for these variables based on all responses.

The questionnaire was elaborated according to the guidelines of the European Statistical System (Eurostat, 2005). After submitting an application to the University Student Council Board (USCB) to obtain permission to complete the study, an online focus-group of five students and one sociologist was organised to discuss the list of questions regarding students’ volunteering drawn from a literature review to develop the standardised questionnaire. The questionnaire was then assessed by four members of the USCB, one physician and one sociologist and pre-tested via an online platform with another five students to reformulate the five questions. After the pilot study, the final version of the questionnaire was evaluated by an additional three external reviewers: two students and one sociologist and received approval from the USCB. Additionally, ethics approval and research governance approval were obtained from the PUMS Bioethics Committee (KB—831/20). Finally, the questionnaire was posted on an online platform and distributed to students engaged in voluntary service. All students received an invitation letter, and informed consent was obtained from all respondents enrolled in the study.

The data collected in the questionnaires were verified and checked for completeness, quality and consistency and exported into the statistical package JASP (Version 0.12.2) for presentation as descriptive statistics. The Mann–Whitney U test was used to compare differences between groups (faculty, study year, gender and religiosity), with a 5% level of significance used for all hypothesis tests. Cronbach’s statistic, alpha, was used as a measure of the internal consistency of the items in the questionnaire. The overall alpha coefficient of the questionnaire was 0.836 (95% CI:0.812–0.858) and was found to be sufficient for the purposes of the study.

## Results

From a total number of 741 PUMS students who engaged in volunteering during the COVID-19 pandemic between 5 May and 30 June, 417 (56.3%) participated in the study. Students, who refused to complete the questionnaire did so because they lacked the time or the interest in the study, were unwilling to discuss their attitudes towards volunteering or quit due to personal reasons. Overall, 72.2% of the respondents were females and 27.8% were males, all of Polish origin (Table 1). The overrepresentation of females can be explained by the fact that medical studies are strongly gendered in Poland, as females accounted for 77.2% of all students at PUMPS (4888 female vs 1442 male students) in 2019. Similarly, females accounted for 54% of all tertiary students, 57.7% of all graduates and 74% in the field of health and welfare in Europe in 2018 (Eurostat, 2020b).

**Table 1:** Students sociodemographic characteristics

Characteristics	*N* (%)
*Gender*	
Female	301 (72.2)
Male	116 (27.8)
*Year of study*	
1	40 (9.6)
2	87 (20.9)
3	56 (13.4)
4	99 (23.7)
5	74 (17.8)
6	61 (14.6)
*Faculty*	
Medicine	256 (61.4)
Nursing	42 (10.1)
Pharmacy	23 (5.5)
Electroradiology	20 (4.8)
Medical analytics	19 (4.5)
Dentistry	14 (3.4)
Midwifery	11 (2.6)
Medical rescue	10 (2.4)
Other	22 (5.3)
*Domicile*	
Up to 10,000 inhabitants	72 (17.3)
10–50,000 inhabitants	64 (15.3)
51–100,000 inhabitants	17 (4.1)
101–500,000 inhabitants	46 (11)
Above 500,000 inhabitants	218 (52.3)
*Confession*	
Christian	273 (65.5)
Agnostic	48 (11.5)
Atheist	96 (23)
*Religious practices*	
Believing/practising	141 (33.8)
Believing /not practising	129 (30.9)
Nonbeliever/practising	6 (1.4)
Nonbeliever/not practising	141 (33.8)
*What role does religion play in your life?*	
Significant, it influences my life decisions and choices	48 (11.5)
Rather big, I try to follow religious principles in my life	93 (22.3)
Little, I separate religion from public issues	137 (32.9)
None, it is irrelevant to me	139 (33.3)

Students who were in the last two of their three years of study volunteered more often than those in their first, second or third year (56.1% vs 43.9%, respectively). The vast majority were students of the medical faculty (61.4%) and lived in large agglomerations (52.3%). Although most respondents declared themselves as Christians (65.5%) and practising believers (33.8%), only 11.5% declared that religion played a significant role in their life.

Based on the responses regarding students’ attachment to religion and its role in their life, it seemed reasonable to divide the respondents into two groups: religious (students who declared that religion influences their life decisions and choices) versus ambivalent/non-religious (students who were not attached to religion or felt it was irrelevant to them). The comparison of these two groups showed that they did not differ in terms of gender, faculty and year of study. The only statistically significant difference was that more students who declared that religion played an important role in their life came from the smallest towns and villages (*p* = 0.021), while students who were ambivalent towards religion lived mainly in large cities (*p* = 0.03).

After hearing the news about the COVID-19 outbreak, both students who were strongly attached to their religion and those who felt ambivalent about it felt the need to act and engage in the fight against the pandemic (64.4% vs 59.1%, respectively) (Table 2). However, the former was statistically significantly more concerned that the pandemic might harm their loved ones (*p* = 0.015), while the latter felt anger more often (*p* = 0.036).

**Table 2:** Students’ experiences during the COVID-19 outbreak

	N (%)		W	*p*
	Religious students	Ambivalent/non-religious students		
What were your feelings after hearing about the COVID-19 outbreak?				
Fear for loved ones	39 (**81.3**)	234 (63.4)	7276.5	**0.015**
Willingness to act	31 (64.4)	218 (59.1)	8368.5	0.465
Fear about my own future	10 (20.8)	105 (28.5)	9531.0	0.267
Anger	11 (22.9)	142 (**38.5**)	10234.5	**0.036**
Nothing, it was irrelevant to me	2 (4.2)	22 (6)	9015.0	0.617
Were you anxious about anything during your voluntary service?				
That I can get infected	15 (31.3)	116 (31.4)	8872.5	0.980
That the healthcare system may collapse	6 (12.5)	126 (**34.1**)	10773.0	**0.002**
That the pandemic will affect my studies	23 (47.9)	175 (47.4)	8812.5	0.950
That the pandemic will affect the situation in the country	19 (39.6)	180 (48.8)	9670.5	0.231
That I will not handle it	6 (12.5)	63 (17.1)	9261.0	0.424
That the pandemic will affect my economic situation	6 (12.5)	108 (**29.3**)	10341.0	**0.014**
I had no worries	6 (12.5)	54 (14.6)	9045.0	0.693
How many times have you volunteered before?				
0	13 (27.1)	105 (28.5)	17519.5	0.843
1	1 (2.1)	25 (6.8)	9271.5	0.207
2	5 (10.4)	57 (15.4)	9301.5	0.358
3-5	16 (33.3)	90 (24.4)	8064.0	0.181
6-10	3 (6.3)	31 (8.4)	9046.5	0.610
>10	10 (20.8)	62 (16.8)	8499.0	0.488
Has volunteering met your expectations?			8412.0	0.424
Yes	40 (83.3)	289 (78.3)		
No	8 (16.7)	80 (21.7)		

Statistically significant differences are written in boldface

When asked about their concerns related to their voluntary service during the COVID-19 outbreak, students who were ambivalent or irrelevant about religion felt more anxious about all the possible dangers related to volunteering and the negative consequences of the COVID-19 pandemic. In particular, they differed in a statistically significant way in their concerns regarding the possibility that the healthcare system might collapse (*p* = 0.002) and that the pandemic would affect their economic situation (*p* = 0.014).

The analysis did not reveal any differences between both groups in their earlier involvement in volunteering, as more than two-thirds of the respondents in both groups had volunteered before. Moreover, neither the differences in the frequency of previous volunteering nor in volunteers’ opinions about their expectations regarding the current voluntary service were found.

Although religion was not a key predictor of the students’ volunteering, it played a significant role in determining their motivations to join the fight against the pandemic. When asked about the reasons that influenced their decision about engaging in voluntary service after COVID-19 struck, the means calculated from the weights assigned differed significantly in the case of eight responses. As shown in Table 3, students who felt strongly attached to their religion were statistically significantly driven by altruistic values more often than those for whom religion was irrelevant. While the former scored much higher when asked about such pro-social reasons as to help others (*p* ≤ 0.001), to give something back to the community (*p* = 0.003), to realise the duty of public service (*p* ≤ 0.001), to help succeed in the fight against the pandemic (*p* = 0.021) and to participate in something important (*p* = 0.006), non-religious students wanted to enhance their professional résumé more often (*p* = 0.041).

**Table 3:** The impact of religion on students’ motivations to volunteer

Motivations	Mean			
	Religious students	Ambivalent/non-religious students	W	*p*
To enhance my professional résumé	1.83	**2.19**	10,377.0	**0.041**
To get new knowledge and skills	3.73	3.68	8441.5	0.585
To gain professional experience	3.54	3.59	8967.0	0.884
To make new contacts that might help me in the future	2.71	2.79	9238.5	0.617
To help others	**4.81**	4.32	6236.0	** < 0.001**
To contribute to the community	**4.52**	4.04	6643.5	**0.003**
To realise the duty of public service inherent to the medical profession	**4.06**	3.37	6327.0	** < 0.001**
To help succeed in the fight against the pandemic	**3.96**	3.60	7103.5	**0.021**
To participate in something important	**4.04**	3.60	6778.0	**0.006**
To have a sense of duty and pride	3.77	3.41	7364.5	0.051
To realise my passion	**4.23**	3.50	5722.0	** < 0.001**
To experience the adventure and to tell my future kids that I was a part of it	2.81	2.69	8492.0	0.636
To fill free time	2.23	**2.98**	11,473.5	** < 0.001**
To make new friends and establish new connections	2.56	2,62	9187.5	0.665
To work with other people	3.58	3.43	8088.5	0.315
To gain the recognition of my professors, family and friends	1.85	1.92	9468.5	0.404

Statistically significant differences are written in boldface

## Discussion and Conclusions

Compared to other European countries, Poland is still a conservative and religious nation, and religion in the country is still highly institutionalised, both at the public and private level. However, Poland is currently experiencing a changing relationship with the Church, as much of the population is moving towards a more secular view of life, one with a greater separation between the Church and the state, and a rejection of Church mandates on individual morality. Consequently, volunteering is also frequently perceived by many as a form of social activism rather than as a religious or moral duty. Thus, even though most Polish volunteers are still driven by altruistic motives, studies show that especially young people, including students, increasingly perceive volunteering as a possibility to gain new knowledge and skills that may benefit their future professional career (Centrum Badania Opinii Społecznej, 2011b*;* Sawicki, 2014; Olczyk, 2015; Jaronowska, 2017; Lubrańska & Zawira, 2017; Główny Urząd Statystyczny, 2017). A similar observation has been made in other countries, where traditional volunteering is being replaced by its modern form (Brooks, 2002; Fényes & Pusztai, 2012; Handy et al., 2010; Holdsworth, 2010; Hustinx, 2001; Hustinx & Lammertyn, 2003; Rehberg, 2005; Voicu & Voicu, 2009).

At the same time, this study shows that similar to other countries, future healthcare professionals in Poland expressed a strong interest in active participation during the current health care emergency (Chawłowska et al., 2021; Drexler et al., 2020; Gallagher & Schleyer, 2020; Gouda et al., 2020; Miller et al., 2020; Soled et al., 2020; Thomson & Lovegrove, 2020). Furthermore, although students’ religiosity was not a significant predictor of volunteering during the COVID-19 outbreak, it influenced their motivations to join the fight against the pandemic. Indeed, while the religious students were driven by altruistic motives (being useful for the society and doing something for others) at a higher rate, the non-religious students were focused more often on building their professional résumé and spending time in a useful way. Moreover, as the former stressed the desire to help others and to give something back to the community, they also more frequently emphasised that nurturance and care for others were deeply embedded in the role of health professionals. Consequently, they wanted to realise the duty of public service and help succeed in the fight against the pandemic. Thus, it seems that particularly during the current health crisis caused by the COVID-19 outbreak, religion strengthened students’ perception of the medical profession as a unique vocation and moral service, often stressing that as future health professionals they felt it was their duty to engage with and help those in need, disregarding the risk. Nevertheless, even in the case of students who defined themselves as deeply religious, pure altruism was not the only motivation, as most respondents volunteered to gain skills, connections or some kind of psychological satisfaction. Thus, it should be acknowledged that the motivations of many students were often a mixture of altruistic and egoistical drivers.

It was also striking that although all the students were concerned about their health and the future of their education, non-religious students were more anxious about the risk associated with the voluntary service itself and possible consequences of the COVID-19 pandemic on society, the country’s economy, the healthcare system and their situation. Moreover, while religiously indifferent students felt anger more often, students who felt attached to their religion feared that the pandemic might harm their loved ones. Thus, again, students’ religiosity was strongly correlated with altruistic values. However, for many volunteers religion served as a unique protective factor that helped with facing and coping with stress, making them feel more competent to volunteer.

To conclude, while this study shows that students’ religiosity was not a significant predictor of volunteering during the COVID-19 pandemic, it did influence differences in students’ motivation. Thus, it shows that all students committed to volunteering during the current health crisis constitute a good example of active citizenship and civic responsibility. At the same time, while both religious and non-religious students had altruistic and self-interested motivations for volunteering, several altruistic motivations were more common among religious students.

### Study Limitations

The findings from this study are limited in several respects that may impact their generalisability and interpretation. First, although the response rate was reasonably high, students from only one Polish medical university were enrolled in the study. Second, as many students refused to complete the questionnaire, the results represent solely the opinions of those who agreed to participate in the study and cannot be generalised for the entire population of future health professionals either in Poznan or in Poland as a whole. Third, as we did not ask specific questions regarding students’ religious attendance and beliefs, further in-depth studies would be required. However, we believe that as this is the first study examining the link between student’s religiosity and motivations to volunteer during a COVID-19 pandemic in Poland it may stimulate further research on the topic**.**

## References

[CR1] Abuiyada R (2018). Students’ Attitudes towards voluntary services: A study of Dhofar University. Journal of Sociology and Social Work.

[CR2] Ariza-Montes A, Tirado-Valencia P, Fernández Rodríguez V, Hager MA (2018). Religious vs secular volunteering motivations: a study on european elders. Research on Ageing and Social Policy.

[CR3] Brooks K (2002). Talking about volunteering: A discourse analysis approach to volunteer motivations. Voluntary Action.

[CR4] Centrum Badania Opinii Społecznej [Centre for Public Opinion Research]. (2011a). Aktywność społeczna Polaków—poziom zaangażowania i motywacje [Social activity the Poles. Level of commitment and motivations]. Retrieved June 25, 2020 from https://www.cbos.pl/SPISKOM.POL/2011/K_062_11.PDF

[CR5] Centrum Badania Opinii Społecznej [Centre for Public Opinion Research]. (2011b). Młody, bogaty, wykształcony, religijny—mit polskiego wolontariusza [The young, the rich, the educated, the religious—the myth of Polish volunteer]. Retrieved December 9, 2020 from https://www.cbos.pl/SPISKOM.POL/2011/K_063_11.PDF

[CR6] Centrum Badania Opinii Społecznej [Centre for Public Opinion Research]. (2012). Potencjał społecznikowski i zaangażowanie Polaków w wolontariat [The public-spirited potential and engagement of the Polish in voluntary service]. Retrieved December 9, 2020 from https://www.cbos.pl/SPISKOM.POL/2012/K_023_12.PDF

[CR7] Chawłowska E, Staszewski R, Lipiak A, Giernas B, Karasiewicz M, ´ Bazan D, Nowosadko M, Cofta M, Wysocki J (2021). Student volunteering as a solution for undergraduate health professions education: Lessons from the COVID-19 pandemic. Frontiers in Public Health.

[CR8] Davie G (2006). Religion in Europe in the 21st century: The factors to take into account. European Journal of Sociology.

[CR9] DeAngelis RT, Acevedo GA, Xu X (2016). Secular volunteerism among Texan emerging adults: Exploring pathways of childhood and adulthood religiosity. Religions.

[CR10] Drexler R, Hambrecht JM, Oldhafer KJ (2020). Involvement of medical students during the coronavirus disease 2019 pandemic: A cross-sectional survey study. Cureus.

[CR11] Einolf C (2011). The link between religion and helping others: The role of values, ideas, and language. Sociology of Religion.

[CR12] Eurostat. (2020a). *Practising physicians*. https://ec.europa.eu/eurostat/databrowser/view/tps00044/default/table?lang=en. Retrieved from 1 July, 2020.

[CR13] Eurostat. Brancato, G., Macchia, S., Murgia, M., Signore, M., Simeoni, G., Blanke, K., Körner, T., Nimmergut, A., Lima, P., Paulino, R., & Hoffmeyer-Zlotnik, J. H. P. (2005). The *Handbook of Recommended Practices for Questionnaire Development and Testing in the European Statistical System*.file:///C:/Users/user/Desktop/RPSQDET27062006.pdf Retrieved from 28 June 2020.

[CR14] Eurostat: Statistics Explained. (2020b). *Tertiary education statistics*. https://ec.europa.eu/eurostat/statistics-explained/index.php/Tertiary_education_statistics#Gender_distribution_of_participation. Retrieved from 9, Dec 2020

[CR15] Fényes H (2015). Effect of religiosity on volunteering and on the types of volunteering among higher education students in a cross-border central and eastern European region. Acta Universitatis Sapientiae, Social Analysis.

[CR16] Fényes H, Pusztai G (2012). Volunteering among higher education students focusing on the micro-level effects on volunteering. Journal of Social Research and Policy.

[CR17] Gallagher T, Schleyer A (2020). “We signed up for this!”—student and trainee responses to the COVID-19 pandemic. New England Journal of Medicine.

[CR18] Główny Urząd Statystyczny [Central Statistical Office]. (2017). *Wolontariat w 2016 r.* [*Volunteering in 2016*] Warszawa: Główny Urząd Statystyczny. https://stat.gov.pl/obszary-tematyczne/gospodarka-spoleczna-wolontariat/wolontariat-i-praca-niezarobkowa-na-rzecz-innych/wolontariat-w-2016-r-,1,3.html. Retrieved from 9, Dec 2020

[CR19] Gouda P, Kirk A, Sweeney AM, O’Donovan D (2020). Attitudes of medical students toward volunteering in emergency situations. Disaster Medicine and Public Health Preparedness.

[CR20] Grönlund H (2012). Religiousness and volunteering: Searching for connections in late modernity. Nordic Journal of Religion and Society.

[CR21] Handy F, Cnaan RA, Hustinx L, Kang C, Brudney JL, Haski-Leventhal D, Holmes K, Meijs LCPM, Pessi AB, Ranade B, Yamauchi N, Zrinscak S (2010). A cross-cultural examination of student volunteering: Is it all about résumé building?. Nonprofit and Voluntary Sector Quarterly.

[CR22] Herzog PS, Strohmeier A, King DP, Khader RA, Williams AL, Goodwin JL, Doan DRH, Moyo B (2020). Religiosity and generosity: Multi-level approaches to studying the religiousness of prosocial actions. Religions.

[CR23] Hill JP, den Dulk KR (2013). Religion, volunteering, and educational setting: The effect of youth schooling type on civic engagement. Journal for the Scientific Study of Religion.

[CR24] Holdsworth C (2010). Why volunteer? Understanding motivations for student volunteering. British Journal of Educational Studies.

[CR25] Hustinx L (2001). Individualism and new styles of youth volunteering: An empirical exploration. Voluntary Action.

[CR26] Hustinx L, Lammertyn F (2003). Collective and reflexive styles of volunteering: A sociological modernization perspective. Society..

[CR27] Hustinx L, Von Essen J, Haers J, Mels S (2015). Religion and volunteering. Complex, contested and ambiguous relationships.

[CR28] Jaronowska S, Kulesza EM, Kosewska B (2017). Przesłanki i uwarunkowania wolontariatu w Polsce [The rationales and determinants of volunteering in Poland]. Wolontariat studencki krajowy i zagraniczny [Students’ volunteering: domestic and foreign].

[CR29] Johnston JB (2013). Religion and volunteering over the adult life course. Journal for the Scientific Study of Religion.

[CR30] Lubrańska A, Zawira E (2017). Motywy współczesnych wolontariuszy w aspekcie różnic pokoleniowych [Motivations of modern volunteers in the light of generational differences]. Łódzkie Studia Teologiczne.

[CR31] Merino SM (2013). Religious social networks and volunteering: Examining recruitment via close ties. Review of Religious Research.

[CR32] Miller DG, Pierson L, Doernberg S (2020). The role of medical students during the COVID-19 pandemic. Annals of Internal Medicine.

[CR33] Monsma SV (2007). Religion and philanthropic giving and volunteering: Building blocks for civic responsibility. Interdisciplinary Journal of Research on Religion.

[CR34] Niebuur J, van Lente L, Liefbroer AC, Steverink N, Smidt N (2018). Determinants of participation in voluntary work: a systematic review and meta-analysis of longitudinal cohort studies. BMC Public Health.

[CR35] Olczyk M (2015). Wolontariat jak forma świadectwa ludzi ochrzczonych [Voluntary service as a form of testimony of the baptised]. Teologia i Moralność.

[CR36] Pawlikowski J, Sak JJ, Marczewski K (2012). Physicians’ religiosity and attitudes towards patients. Annals of Agricultural and Environmental Medicine.

[CR37] Paxton P, Reith NE, Glanvill JL (2014). Volunteering and the dimensions of religiosity: A cross-national analysis. Review of Religious Research.

[CR38] Petrovic K, Stukas AA, Marques MD (2020). Religiosity, motivations, and volunteering: A test of two theories of religious prosociality. Journal of Theoretical Social Psychology.

[CR39] Rasmussen S, Sperling P, Poulsen MS, Emmersen J, Andersen S (2020). Medical students for health-care staff shortages during the COVID-19 pandemic. Lancet.

[CR40] Rehberg W (2005). Altruistic individualists: Motivations for international volunteering among young adults in Switzerland. Voluntas International Journal of Voluntary and Nonprofit Organizations.

[CR41] Sawicki R (2014). Religijność młodzieży zaangażowanej w wolontariat szkolnych kół Caritas w diecezji ełckiej w świetle badań empirycznych [Religiosity of the youth engaged in school Caritas clubs in Ełk diocese in the light of empirical research]. Studia Ełckie.

[CR42] Soled D, Goel S, Barry D, Erfani P, Joseph N, Kochis M, Uppal N, Velasquez D, Vora K, Scott KW (2020). Medical student mobilization during a crisis: Lessons from a COVID-19 medical student response team. Academic Medicine.

[CR43] Stokes DC (2020). Senior medical students in the COVID-19 response: An opportunity to be proactive. Academic Emergency Medicine.

[CR44] Szluz B (2012). Wolontariat studentów—współczesne wyzwania [Students’ volunteerism—modern challenges]. Studia Socialia Cracoviensia.

[CR45] Szymańska-Palaczyk, A. (2016). Wolontariat w Polsce. Przykład projektu Polska Cyfrowa Równych Szans [*Voluntary service in Poland*. *An example of the project Digital Poland Equal Opportunities*]. *Ruch Prawniczy, Ekonomiczny i Socjologiczny*, *78*(1), 255–271.

[CR46] Thomson E, Lovegrove S (2020). ‘Let us help’—why senior medical students are the next step in battling the COVID-19 pandemic. International Journal of Clinical Practice.

[CR47] Voicu B, Voicu M (2009). Volunteers and volunteering in Central and Eastern Europe. Sociológia.

[CR48] Wenger NS, Carmel S (2004). Physicians' religiosity and end-of-life care attitudes and behaviors. Mount Sinai Journal of Medicine.

[CR49] Wilson J, Janoski T (1995). The contribution of religion to volunteer work. Sociology of Religion.

[CR50] Yeung JWK (2018). Are religious people really more helpful? Public and private religiosity and volunteering participation. Nonprofit and Voluntary Sector Quarterly.

